# Disseminated Intravascular Coagulation and Malignancy: A Case Report and Literature Review

**DOI:** 10.1155/2020/9147105

**Published:** 2020-01-02

**Authors:** Sumit Sohal, Akhilesh Thakur, Aleena Zia, Mina Sous, Daniela Trelles

**Affiliations:** Department of Internal Medicine, AMITA Health Saint Francis Hospital, 355 Ridge Avenue, Evanston, IL 60202, USA

## Abstract

Disseminated Intravascular Coagulation (DIC) is a disorder of coagulation which is commonly seen as a complication of infections, traumas, obstetric diseases, and cancers especially hematological and rarely solid cancers. DIC may rarely be the presenting feature of an undiagnosed malignancy. It may present in the form of different phenotypes which makes its diagnosis difficult and leads to high mortality. The treatment comprises supportive, symptomatic treatment and removal of the underlying source. Here, we present a patient with history of being on warfarin for atrial fibrillation and other comorbidities who presented with elevated INR of 6.3 and increasing dyspnea on exertion. Over the course of her stay, her platelet counts started dropping with a concurrent decrease in fibrinogen levels. She eventually developed pulmonary embolism, followed by stroke and limb ischemia, which was indicative of the thrombotic phenotype of DIC. Her pleural fluid analysis showed huge burden of malignant cells in glandular pattern suggestive of adenocarcinoma and was started on heparin drip. However, the patient had cardiac arrest and expired on the same day of diagnosis.

## 1. Introduction

Disseminated Intravascular Coagulation (DIC) is a condition characterized by systemic activation of coagulation, potentially leading to thrombotic obstruction of small and midsize vessels, thereby contributing to organ dysfunction [1]. It may result as a complication of infection, solid cancers, hematological malignancies, obstetric diseases, trauma, aneurysms, liver diseases, etc. [2]. DIC reportedly develops in 10% to 15% of patients with cancer where it may exhibit a chronic phenotype or present as an acute phenomenon [3]. We present a case of a patient who was admitted with unrelated complaints and developed DIC during her hospital course, with her workup revealing an underlying malignancy.

## 2. Case Presentation

An 81-year-old female with a past medical history of hypertension, hyperlipidemia, orthostatic hypotension, atrial fibrillation, and bioprosthetic mitral valve replacement on warfarin presented at clinic after the International Normalized Ratio (INR) was reported as 6.3, without any signs or symptoms of bleeding. On review of systems, the patient endorsed increased dyspnea on exertion over the last few weeks. She did not endorse a change in dose or diet and no new medications except for methylprednisolone (Medrol) dose pack about a week prior to admission.

Physical exam on presentation was significant for a temperature of 97.4°F, blood pressure of 130/70, heart rate of 83 beats per minute (bpm), and an oxygen saturation of 96% on room air. She had decreased breath sounds at lung bases and superficial scratches on bilateral upper extremities with no overt signs of bleeding. Laboratory findings were significant for white blood count of 13.5 k/mm cu (ref range: 4.0-11.0 k/mm cu), hemoglobin 10.8 g/dl (ref range: 12-15.3 g/dl), and platelet count 285 k/cu mm (ref range: 150-450 k/cu mm). Chest X-ray showed minimal increased opacity at the left lung base, a nodular opacity in the right midlung unchanged from the prior study, and slight blunting of the costophrenic angles bilaterally. CT chest without contrast was done and showed chronic diffuse emphysema, minimal bilateral pleural effusion, and a reidentified spiculated left upper lobe mass that measured 0.7 × 0.6 cm which had slightly increased in conspicuity (previously 0.4 × 0.5 cm). Warfarin was held and IV Vitamin K 5 mg was given. INR dropped to 1.4 the next day, and the patient was started on low-dose warfarin. She was also started on empiric antibiotics (vancomycin and piperacillin/tazobactam) as she showed worsening respiratory status.

**Figure 1:**
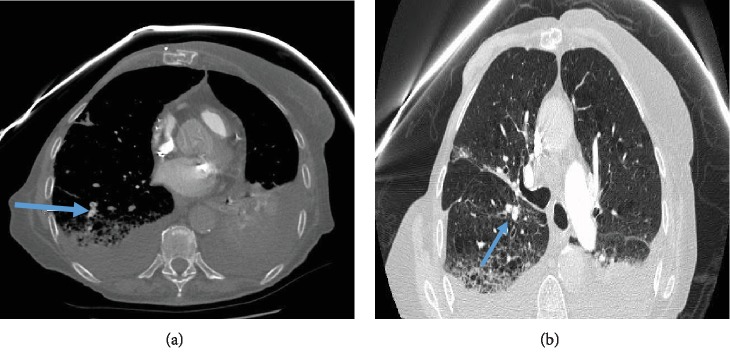
(a, b) A CT angiogram of the chest shows filling defects in the subsegmental arteries (arrows).

**Figure 2:**
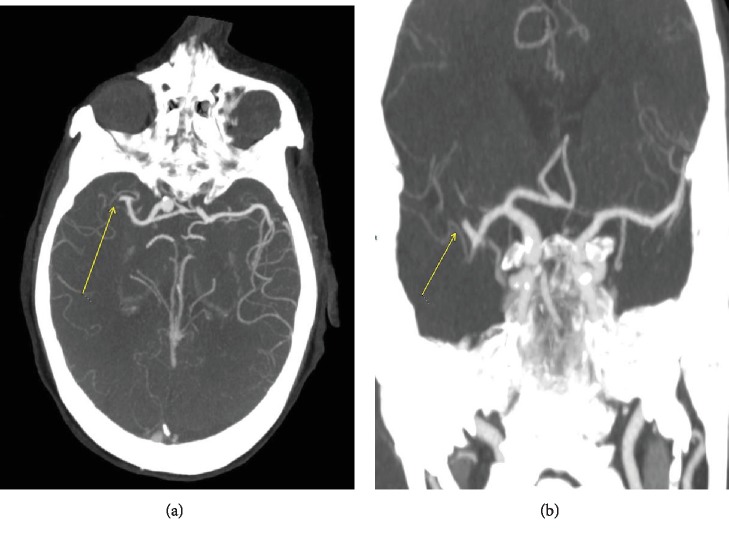
(a, b) CT angiogram of the head shows abrupt occlusion of the branch of the middle cerebral artery (arrows).

Her platelets on the 3^rd^ day of admission dropped by >50% to 127. She had no exposure to heparin or its products, and her warfarin was held. A peripheral blood film showed no schistocytes. Her hemoglobin dropped to 7 g/dl. Her fibrinogen was 147 mg/dl (ref range: 163-463 mg/dl), INR 1.3, haptoglobin 86 mg/dl (ref range: 44-215 mg/dl), LDH 593 IU/l (ref range: 140-271 IU/l), and total bilirubin 0.4 mg/dl (ref range: 0.0-1.0 mg/dl). Hematology was consulted, and various other tests to exclude multiple diagnoses were initiated. Transthoracic echocardiography was done and showed an ejection fraction of 59% and ruled out any vegetation on valves. Her blood cultures and urine cultures remained negative. Her platelets continued to drop. On the 8^th^ day of admission, her respiratory status continued to worsen, and hence, CT angiogram of the chest was done which revealed multiple subsegmental pulmonary emboli (SSPE) (Figures 1(a) and 1(b)) and significant pleural effusion (no anticoagulation given as SSPE and platelet level 22 k/cu mm). On the next day, interventional radiology- (IR-) guided thoracentesis was done (after platelet transfusion) and drained 920 cc of sanguineous fluid. This was complicated by pneumothorax, followed by chest tube placement. Later on the same day, her mental status declined, as she appeared confused with slurring of speech and had rightward gaze preference. CT head was done followed by CT angiogram of the head and neck which demonstrated right MCA, M1 occlusion (Figures 2(a) and 2(b)), but she was not a candidate for tissue plasminogen (tPA) due to low platelet count and thrombectomy was not pursued due to her multiple comorbidities. She was then conservatively managed in the ICU.

**Figure 3:**
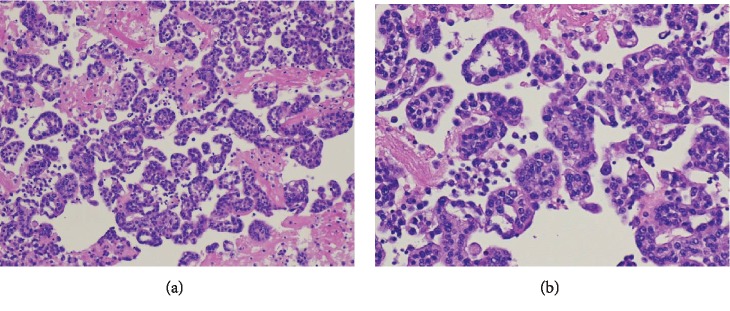
(a, b) Microscopic picture of pleural fluid analysis (200x/400x). Numerous tumor cells arranged in well-differentiated glandular pattern consistent with metastatic adenocarcinoma.

On the 10^th^ day, her lung fluid cytology revealed multiple malignant cells arranged in a well-defined glandular pattern consistent with adenocarcinoma (Figure 3), and simultaneously, it was reported that her left lower extremity turned blue concerning for acute limb ischemia. Her laboratory values of fibrinogen and INR also worsened (87 mg/dl and 1.7, respectively), and she was started on heparin drip for the diagnosis of DIC with multiple thrombotic events secondary to occult malignancy. Unfortunately, the patient had a cardiac arrest and expired that day. The tumor cells came back positive for TTF-1 and negative for Napsin, GATA-3, Calretinin, and CK 5/6, consistent with metastatic adenocarcinoma of lung primary.

## 3. Discussion

DIC can be defined as a widespread hypercoagulable state that can lead to both microvascular and macrovascular clotting and compromised blood flow, ultimately resulting in multiple organ dysfunction syndrome (MODS) [4]. At the same time, the use and subsequent depletion of platelets and coagulation proteins resulting from the ongoing coagulation may induce severe bleeding [5]. In addition to coagulation activation, fibrinolytic activation occurs and the degree of activation of this system leads to the classification of the two major types of symptoms in DIC, i.e., bleeding symptoms and organ symptoms (dysfunction) [6]. When dealing with patients with cancer-related DIC, it is useful to consider the different pathogenic mechanisms that can lead to the abovementioned different clinical manifestations [7].

### 3.1. Diagnosis of DIC

The active members of the subcommittee for DIC of the Scientific and Standardization Committee (SSC) of the International Society on Thrombosis and Haemostasis (ISTH) attempted to harmonize the three guidelines for DIC that have been published in the literature from the British Committee for Standards in Haematology (BCSH), the Japanese Society of Thrombosis and Hemostasis (JSTH), and the Italian Society for Thrombosis and Hemostasis (SISET). Though DIC does not have any gold standard test, ISTH favors the use of scoring systems to diagnose DIC [8–11].

Three different diagnostic scoring systems have been devised by the ISTH/SSC [12], Japanese Ministry Health, Labour and Welfare (JMHLW) [13], and Japanese Association of Acute Medicine (JAAM) [14]. A prospective study in Japan reported no significant differences in the odds ratio for predicting DIC outcomes among these three diagnostic criteria [15]. The bleeding type of DIC can be easily diagnosed using the ISTH overt-DIC and JMHLW criteria, while the organ failure type of DIC is diagnosed according to the JAAM diagnostic criteria [2].

Our patient was positive for DIC as per these criteria as her platelet count (26 k/cu mm), fibrinogen level (87 mg/dl), and INR (1.7) were conclusive for DIC.

### 3.2. DIC and Cancer

Professor Trousseau in 1865 first described an association between recurrent migratory thrombophlebitis and cancer, and since then, several studies have proven a tight relationship between thrombosis and malignant disease. However, the thrombotic complications of cancer are not limited to venous thromboembolism and may appear as DIC [16, 17].

Cancer-related DIC may present in one of these 3 forms: firstly, the “procoagulant” form, where excess thrombin generated causes thrombosis in microvascular and macrovascular fields and thus clinically manifest as DIC with thrombotic features; secondly, the “hyperfibrinolytic” form, where activation of the fibrinolytic system dominates the picture and thus clinically presents as bleeding; and last but not the least, the “subclinical” form, where the amounts of thrombin and plasmin generated do not cause obvious clinical manifestations but can be reflected in laboratory markers of coagulation or fibrinolysis activation. While most of the solid tumors come under the umbrella of subclinical DIC, pancreatic and lung adenocarcinomas are procoagulant whereas acute promyelocytic leukemia and metastatic prostate cancer are likely to be in the hyperfibrinolytic form [7]. Though the association between solid cancers and DIC is well-known and has been described in almost all histologic types [18], the mortality rate from DIC is higher in patients with lung cancer than in non-lung cancer patients and prevalence of DIC varies among the pathologic types of lung cancer [19]. Many studies have shown that patients with adenocarcinoma are at a very high risk of DIC [7] especially in lung adenocarcinoma [20] due to the procoagulant mucin in the cancer cells [21]; however, one recent study has refuted this claim [19].

Our patient with evidence of pulmonary embolism, stroke, and acute limb ischemia presented with thrombotic features of DIC. There was no biopsy performed; however, the malignant cells found on the pleural fluid analysis were consistent with metastatic adenocarcinoma of lung primary.

### 3.3. Treatment of Cancer-Related DIC

The cornerstone of DIC treatment is providing treatment for the underlying disorders, such as the administration of antibiotics or surgical drainage in patients with infectious diseases and anticancer drugs or surgery in patients with malignant diseases [2]. However, patients with DIC resulting from sepsis, hematologic malignancy, or obstetric disease can be successfully treated for DIC, whereas DIC associated with solid cancers may not respond to standard treatments [11].

#### 3.3.1. In Patients with Procoagulant Form of DIC Which Is Characterized by Evidence of Venous or Arterial Thromboembolism with No Evidence of Bleeding

Feinstein [22] recommended to initiate anticoagulant therapy with unfractionated heparin and maintain fibrinogen levels at ≥100 mg/l and platelet counts >50,000/*μ*l via appropriate transfusions of cryoprecipitate and platelets [22]. Wada et al. also recommended therapeutic doses of heparin in cases of DIC where thrombosis predominates [11].

#### 3.3.2. In Patients with Bleeding Form of DIC Which Is Characterized by Severe Bleeding

Wada et al. [11] recommend transfusion of platelets if platelet count < 50 × 10^9^ L^−1^ or in those with a high risk of bleeding and a platelet count of <20 × 10^9^ L^−1^, administration of Fresh Frozen Plasma (FFP) for either prolonged prothrombin time/activated partial thromboplastin time (>1.5 times normal) or decreased fibrinogen (<1.5 g dL^−1^) and fibrinogen concentrate or cryoprecipitate in actively bleeding patients with persisting severe hypofibrinogenemia (<1.5 g L^−1^) despite FFP replacement. Thachil et al. also supported these recommendations [7].

#### 3.3.3. Role of Chemotherapy

The patients with DIC should be started on chemotherapy as soon as clinical problems associated with DIC are under control [22]. No Randomized Control Trials (RCTs) have ever been done for solid tumors; however, several case reports suggest the role of chemotherapy in these patients. Targeted chemotherapies have been used in some case reports with good outcomes [3].

Our patient was on warfarin prior to presentation, and when warfarin was stopped, her platelets started to drop and she initially developed a picture of subclinical DIC, followed by procoagulant form of DIC leading to thrombosis of various organ systems. This happened when her diagnosis was unclear, and eventually when the diagnosis was made, she was well beyond recovery.

## 4. Conclusion

Early diagnosis and identification of underlying condition is the key to reduce mortality in these patients with DIC. It can often be challenging to establish the underlying cause, especially if the patient is on anticoagulants or has other risk factors for bleeding/thrombosis. Malignancy should always be considered in laboratory findings of subclinical DIC, especially without overt cause and with risk factors for lung cancer.
